# A novel tyrosine kinase inhibitor AMN107 (nilotinib) normalizes striatal motor behaviors in a mouse model of Parkinson’s disease

**DOI:** 10.3389/fncel.2014.00050

**Published:** 2014-02-20

**Authors:** Akie Tanabe, Yukio Yamamura, Jiro Kasahara, Ryoma Morigaki, Ryuji Kaji, Satoshi Goto

**Affiliations:** ^1^Department of Motor Neuroscience and Neurotherapeutics, Institute of Health Biosciences, Graduate School of Medical Sciences, University of TokushimaTokushima, Japan; ^2^Department of Neurobiology and Therapeutics, Institute of Health Biosciences, Graduate School of Pharmaceutical Sciences, University of TokushimaTokushima, Japan; ^3^Department of Clinical Neuroscience, Institute of Health Biosciences, Graduate School of Medical Sciences, University of TokushimaTokushima, Japan

**Keywords:** nilotinib, c-Abl inhibitor, Cdk5, DARPP-32, Parkinson’s disease

## Abstract

Abnormal motor behaviors in Parkinson’s disease (PD) result from striatal dysfunction due to an imbalance between dopamine and glutamate transmissions that are integrated by dopamine- and cAMP-regulated phosphoprotein of 32 kDa (DARPP-32). c-Abelson tyrosine kinase (c-Abl) phosphorylates cyclin-dependent kinase 5 (Cdk5) at Tyr15 to increase the activity of Cdk5, which reduces the efficacy of dopaminergic signaling by phosphorylating DARPP-32 at Thr75 in the striatum. Here, we report that in the mouse striatum, a novel c-Abl inhibitor, nilotinib (AMN107), inhibits phosphorylation of both Cdk5 at Tyr15 and DARPP-32 at Thr75, which is negatively regulated by dopamine receptor activation through a D2 receptor-mediated mechanism. Like a D2-agonist, nilotinib synergizes with a D1-agonist for inducing striatal c-Fos expression. Moreover, systemic administration of nilotinib normalizes striatal motor behaviors in a mouse model of PD induced by 1-methyl-4-phenyl-1,2,3,6-tetrahydropyridine. These findings suggest that nilotinib could possibly serve as a new and alternative agent for treating PD motor symptoms.

## INTRODUCTION

**FIGURE 1:**
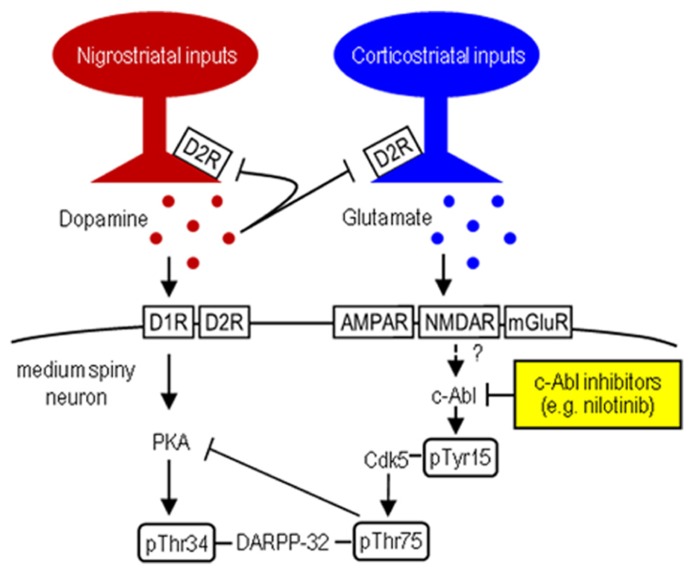
**Striatal Cdk5/DARPP-32 signal regulation.** Hypothesized model shows striatal dopamine and glutamate neurotransmissions involving postsynaptic dopamine/PKA/DARPP-32-pThr34 and glutamate/Cdk5/DARPP-32-pThr75 signaling cascades. c-Abl inhibitors (e.g., nilotinib) target c-Abl that can activate Cdk5 by phosphorylating it at Tyr15.

Striatal dopamine deficiency caused by the degenerative loss of nigral dopaminergic cells is the main pathological feature of Parkinson’s disease (PD; Olanow and Tatton, 1999). The pathophysiology of PD involves dysfunction of the striatum due to an imbalance between dopamine and glutamate transmission that have opposing physiological effects (Greengard, 2001). A key regulator in the integration of dopamine and glutamate is DARPP-32, the dopamine- and cAMP-regulated phosphoprotein of 32 kDa. DARPP-32 is a striatal-enriched phosphoprotein, which can act as either a protein phosphatase inhibitor or a protein kinase inhibitor, depending on whether Thr34 or Thr75 is phosphorylated (Greengard, 2001). Corticostriatal glutamate inputs activate cyclin-dependent kinase 5 (Cdk5), which inhibits postsynaptic dopamine signaling by phosphorylating DARPP-32 at Thr75 (Thr75-DARPP-32) in the striatum (for reference, see **Figure 1**). DARPP-32 with Thr75 phosphorylation (DARPP-32-pThr75) functions as an inhibitor of cAMP-dependent protein kinase A (PKA), a key regulator of dopamine D1 receptor (D1R)-mediated signals. Given the evidence that under resting conditions, striatal Thr75-DARPP-32 is very highly phosphorylated, but striatal Thr34-DARPP-32 is only slightly phosphorylated (Greengard, 2001; Sako et al., 2010), it has been suggested that tonic activity of glutamate/Cdk5 signaling might be responsible for maintaining Thr75-DARPP-32 in a phosphorylated state, thereby inhibiting D1R/PKA signaling in the striatum (Greengard, 2001). Interestingly, in rodent models of PD, striatal dopamine deficiency has been shown to have no effect on phosphorylation of Thr34-DARPP-32, but significantly increase that of Thr75-DARPP-32 (Brown et al., 2005; Santini et al., 2007). These findings suggest that the glutamate/Cdk5/DARPP-32-pThr75 pathway might be important in assessing the molecular mechanisms underlying PD symptoms.

c-Abelson tyrosine kinase (c-Abl) is the prototypic non-receptor tyrosine kinase that is implicated in various cellular processes (Dhavan and Tsai, 2001; Hantschel and Superti-Furga, 2004). Of particular interest is that c-Abl phosphorylates Cdk5 at Tyr15 to increase Cdk5 activity (Zukerberg et al., 2000; Dhavan and Tsai, 2001; Zhang et al., 2007). We recently found that in mice, Cdk5-pTyr15 is highly concentrated in the striatum, particularly in its matrix compartment (Morigaki et al., 2011; Yamamura et al., 2013), where dopamine receptor activation negatively regulates phosphorylation of Tyr15-Cdk5 through a D2R-mediated mechanism (Yamamura et al., 2013). Moreover, in the 1-methyl-4-phenyl-1,2,3,6-tetrahydropyridine (MPTP) mouse model of PD, the c-Abl inhibitor imatinib (STI-571) reverses abnormally increased striatal phosphorylation of Tyr15-Cdk5 and Thr75-DARPP-32, as does L-3,4-dihydroxyphenylalanine (L-DOPA; Yamamura et al., 2013). These findings suggest a new hypothesis that c-Abl signaling might be implicated in striatal function and raise the possibility that a c-Abl inhibitor might inhibit Cdk5/DARPP-32-pThr75 signaling, thereby serving as a tool in treating PD symptoms. In this study, we report that nilotinib (formerly known as AMN107), a second-generation tyrosine kinase inhibitor that targets c-Abl (Weisberg et al., 2006; Blay and von Mehren, 2011), can normalize motor impairments caused by striatal dopamine deficiency in MPTP-treated mice.

## MATERIALS AND METHODS

### ANIMALS

Male C57Bl/6 mice (Japan SLC Co., Shizuoka, Japan) aged 7–8 weeks were used in this study. The mice were housed in a controlled environment (25 ± 1°C, 50 ± 10% humidity, and 12-h light/dark cycle) with access to food and tap water *ad libitum*. All procedures involving experimental mice were approved by the Ethical Review Committee of the University of Tokushima.

### NILOTINIB ADMINISTRATION

Mice received single intraperitoneal injections of nilotinib (5, 10, 25, or 50 mg/kg; Cayman Chemical, Ann Arbor, MI, USA) dissolved in 0.5% aqueous carboxymethyl cellulose 3 days after administration of MPTP or saline. Vehicle-treated (control) mice received an equivalent volume of 0.5% aqueous carboxymethyl cellulose.

### ADMINISTRATION OF DOPAMINERGIC DRUGS

In combination with nilotinib (25 mg/kg), A-68930 hydrochloride (2 mg/kg of free base; Sigma-Aldrich, St Louis, MO, USA), SCH-23390 hydrochloride (0.5 mg/kg of free base; Sigma-Aldrich), quinpirole hydrochloride (3 mg/kg of free base; Sigma-Aldrich), or raclopride hydrochloride (1 mg/kg of free base; Sigma-Aldrich) was dissolved in saline and intraperitoneally injected 30 min before sacrifice. The dose of each drug was determined as described previously (Yamamura et al., 2013).

### MPTP ADMINISTRATION

Mice were injected intraperitoneally four times in a day with MPTP hydrochloride (20 mg/kg of free base; Sigma-Aldrich) at 2-h intervals (Aoki et al., 2009; Yamamura et al., 2013). Saline-treated mice received an equivalent volume of 0.9% saline. Our previous study showed that maximal neurodegenerative effects of MPTP on the nigral dopaminergic cells were observed at the 3-day time-point after administration of MPTP (Aoki et al., 2009; Yamamura et al., 2013).

### WESTERN BLOT ANALYSIS

Striatal tissue samples were homogenized in ice-cold lysis buffer containing 50 mM Tris–HCl buffer, pH 7.5, with 0.5 M NaCl, 0.5% Triton X-100, 10 mM EDTA (ethylenediaminetetraacetic acid), 4 mM EGTA (ethylene glycol tetraacetic acid), 1 mM Na_3_VO_4_, 30 mM Na_2_P_2_O_7_, 50 mM NaF, 0.1 mM leupeptin, 75 μM pepstatin A, 50 μg/ml trypsin inhibitor, 1 mM phenylmethanesulfonyl fluoride, 100 nM calyculin A, and 1 mM dithiothreitol. After centrifugation at 15,000 rpm for 10 min at 4°C, the protein lysates were mixed with Laemmli’s buffer containing 63 mM Tris–HCl, pH 6.8, 2% sodium dodecyl sulfate (SDS), 5% 2-mercaptoethanol, 2.5% glycerol, and 0.01% bromophenol blue, and were then heated at 100°C for 5 min. Each sample, containing the same amount of protein, was applied to a 10% SDS-polyacrylamide gel for electrophoresis (PAGE) followed by blotting onto a PVDF (polyvinylidene fluoride) membrane. The PVDF membranes were then incubated with the desired primary antibodies. Antibodies against tyrosine hydroxylase (TH; 1:1000; Millipore, Billerica, MA, USA), Cdk5-pTyr15 (1:1000; Santa Cruz Biotechnology, Santa Cruz, CA, USA), Cdk5 (1:1000; Santa Cruz Biotechnology), and DARPP-32 (1:1000; Cell Signaling, Danvers, MA, USA) DARPP-32-pThr34 (1:1000; Cell Signaling), and DARPP-32-pThr75 (1:1000; Cell Signaling) were used. Anti-β-actin antibody (1:5000; Sigma-Aldrich) was used to adjust for equal amounts of protein loading into each well. The bound antibodies were detected by the ECL (enhanced chemiluminescence) method with horseradish peroxidase-conjugated secondary antibodies. Gel images were captured using a lumino-imaging analyzer LAS-4000 (Fujifilm, Tokyo, Japan). Optical densities were evaluated using a computerized image analysis system (Dolphin-DOC; Kurabo, Osaka, Japan).

### TISSUE PREPARATION AND c-Fos IMMUNOSTAINING

Mice were injected intraperitoneally with a lethal dose of pentobarbital (Sigma-Aldrich) 120 min after drug administration. They were then transcardially perfused with 0.01 M phosphate-buffered saline (PBS) at pH 7.4, followed by cold 4% paraformaldehyde in 0.1 M phosphate buffer (PB) at pH 7.4. The brains were removed, post-fixed overnight in the same fixative at 4°C, and moved through a 10–30% sucrose gradient in 0.1 M PB at 4°C for cryoprotection. Sections were cut on a cryostat at 15-μm thickness, and stored in PBS containing 0.05% NaN_3_ until use. Free-floating brain sections were incubated in PBS containing 3% bovine serum albumin (BSA) and rabbit polyclonal antibody to c-Fos (1:50,000; Oncogene Science, Cambridge, MA, USA) at room temperature overnight. The bound primary antibodies were detected by the Histofine Simple Stain Kit (Nichirei, Tokyo, Japan) and the TSA (tyramide signal amplification) system with Cyanine3 (PerkinElmer; Shelton, CT, USA; Goto et al., 2013; Koizumi et al., 2013).

### DIGITAL IMAGING AND NUCLEAR DENSITOMETRY

Digital microscopic images were captured using an Olympus BX51 microscope (Olympus, Tokyo, Japan), imported into Adobe Photoshop CS4, and processed digitally for adjustments of contrast, brightness, and color balance. Measurements of the density of c-Fos-labeled nuclei were made on the striatal sections at the level of 0.9–1.1 mm anterior to bregma, according to the atlas of Hof et al. (2000). We counted the number of c-Fos-positive nuclei in five striatal fields of each mouse (*n* = 5), and calculated the density of c-Fos-positive nuclei/mm^2^ in each animal (Sako et al., 2010).

### BEHAVIORAL TESTS

Prior to pharmacological testing, mice were handled for a week by the same operator to reduce stress, and trained for behavioral tests as described below until their motor performance became reproducible. All behavioral training and tests were performed by the same operator from 10:00 to 16:00 in the order to lower stress. The experimental room environment was kept constant for all tests. Apparatus was cleaned with 70% ethanol on each trial.

#### Beam walking test

This test evaluates motor coordination and balance in rodents. The testing apparatus consists of a rough round horizontal beam (wood, 8 mm diameter for test trial or 16 mm for training trial, 80 cm length) fixed 60 cm above a countertop, and a dark goal box (15 cm width, 10 cm length, 10 cm height). Mice were trained to traverse the beam without stopping on the way for three consecutive days before MPTP administration. In test trials, mice were made to traverse the beam in the same manner (cut-off time 60 s maximum). The traveling time from the start to the 50 cm point was recorded.

#### Bar test

This test is also known as the catalepsy test, and it estimates the ability of an animal to escape from an externally imposed posture. The testing apparatus consists of a horizontal metal bar (3 mm diameter) fixed 4 cm above a countertop. The forepaws of mice were gently placed on the bar, and the time for which mice maintained this abnormal posture was measured (cut-off time 120 s maximum). Training trial was performed once a day for three consecutive days before MPTP administration. Testing trials were performed in the same manner.

#### Horizontal wire test

This test estimates motor coordination and muscle relaxation. The testing apparatus consists of a solid wire (1 mm diameter, 20 cm length) horizontally stretched 20 cm above a countertop. Mice were lifted by the tail and allowed to grasp the wire with their forepaws, and then were released. A mouse passed this test if it grasped the wire with at least one hindpaw within 3 s. Mice were trained to pass the test for three consecutive days before MPTP administration. The test trial was performed once in the same manner. The rate of mice passing was recorded.

#### Rotarod test

This test evaluates motor coordination and motor learning. The Rota-Rod Treadmill (Constant Speed Model, Ugo Basile, Varese, Italy) was used. On the day before the training session started, mice were habituated to the apparatus for 15 min. In training trials, mice were trained to run on the rotarod (20 rpm) for 10 min without falling, twice a day for three consecutive days before MPTP administration. In test trials, mice were made to run the rod rotating at 28 rpm (cut-off time 600 s maximum). The latency time to fall was recorded.

#### Foot printing test

This test provides information on locomotor gait. The testing apparatus is made of a gray acrylic board (3 mm thick), and consists of a runway (10 cm width, 60 cm length, 12 cm height) with non-slippery white paper and a dark goal box (16 cm width, 10 cm length, 12 cm height). On the first training day, mice were habituated to the apparatus for 2 min, then their forepaws and hindpaws were painted red and green with non-toxic food dyes and trained to run to the goal box (training trial). A training trial was performed once a day for two consecutive days before MPTP administration. In test trials, mice were made to run the runway in the same manner (cut-off time 60 s maximum). The footprint patterns were analyzed for three parameters (stride length, stride width, and overlap), prints near the start and the goal being excluded because of the effects of acceleration or deceleration. Stride length was measured as the average distance between each forepaw and hindpaw footprint. Stride width was measured as the average distance between the right and left footprint of each forepaw and hindpaw. Overlap was measured as the average distance between the center of forepaw and hindpaw footprints on the same side. At least four values were measured in each trial for each parameter.

### STATISTICAL ANALYSIS

All experimental values were expressed as means ± SEM. Statistical significance was evaluated by unpaired two-tailed *t*-test, or by one-way analysis of variance (ANOVA) followed by Newman–Keuls, Scheffe, or Fisher’s PLSD (protected least significant difference) *post hoc* test for pairwise comparisons. The significance level was set at *P* < 0.05. All analyses were conducted in Stat View 5.0 (SAS Institute, Cary, USA).

## RESULTS

### NILOTINIB INHIBITS STRIATAL PHOSPHORYLATION OF Tyr15-Cdk5 AND Thr75-DARPP-32 IN NAÏVE MICE

**FIGURE 2:**
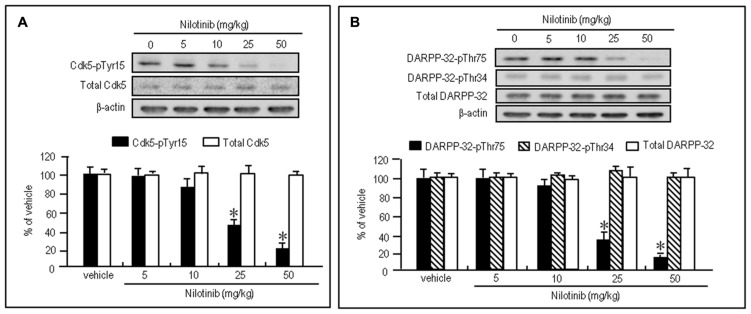
**Nilotinib affects striatal phosphorylation of Cdk5 and DARPP-32 in naïve mice.** Western blot analysis was performed on striatal extracts from naïve mice that received intraperitoneal injections of nilotinib at doses of 5, 10, 25, or 50 mg/kg 30 min before they were sacrificed. **(A)** Optical density measurements of striatal Cdk5-pTyr15 and total Cdk5. Values are means ± SEM (*n* = 5). *P < 0.05 versus mice treated with vehicle; one-way ANOVA [*F*_(4,20)_ = 22.957] followed by Scheffe test. **(B)** Optical density measurements of striatal DARPP-32-pThr75, DARPP-32-pThr34, and total DARPP-32. Values are means ± SEM (*n* = 5). *P < 0.05 versus mice treated with vehicle; one-way ANOVA [*F*_(4,20)_ = 93.125] followed by Scheffe test.

To test whether nilotinib could affect striatal phosphorylation of Cdk5 and DARPP-32, we first performed western blot analysis on the striatal extracts from mice that received intraperitoneal injections of vehicle or nilotinib at doses of 5, 10, 25, or 50 mg/kg 30 min before they were sacrificed. Striatal levels of Cdk5-pTyr15 (**Figure 2A**; *P* < 0.05, ANOVA) and DARPP-32-pThr75 (**Figure 2B**; *P* < 0.05, ANOVA) were significantly reduced in mice injected with nilotinib at the doses of 25 and 50 mg/kg, compared to mice treated with vehicle. In contrast, no effects of nilotinib on striatal levels of total Cdk5 (**Figure 2A**; *P* > 0.05, ANOVA), DARPP-32-pThr34 (**Figure 2B**; *P* > 0.05, ANOVA), and total DARPP-32 (**Figure 2B**; *P* > 0.05, ANOVA) were found. Thus, systemic administration of nilotinib could inhibit striatal phosphorylation of both Cdk5 at Tyr15, the substrate site targeted by c-Abl (Zukerberg et al., 2000; Dhavan and Tsai, 2001; Zhang et al., 2007), and DARPP-32 at Thr75, the substrate site targeted by Cdk5 (Greengard, 2001), in naïve mice.

**FIGURE 3:**
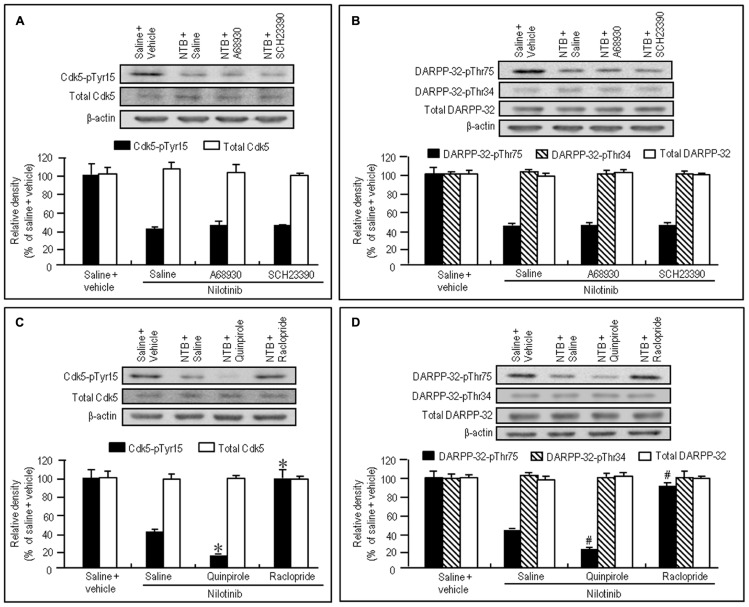
**Dopaminergic modulation of nilotinib effects on striatal phosphorylation of Tyr15-Cdk5 and Thr75-DARPP-32 in naïve mice.** Vehicle or nilotinib (NTB; 25 mg/kg) treated mice were administered saline, A-68930 (2 mg/kg), SCH-23390 (0.5 mg/kg), quinpirole (3 mg/kg), or raclopride (1 mg/kg) 30 min before sacrifice. Striatal tissue extracts were prepared and subjected to western blotting. **(A)** Western blot analysis of striatal levels of Cdk5-pTyr15 and total Cdk5 in mice treated with saline + vehicle, NTB + saline, NTB + A-68930, or NTB + SCH-23390. Values are means ± SEM (*n* = 5). **(B)** Western blot analysis of striatal levels of DARPP-32-pThr75, DARPP-32-pThr34, and total DARPP-32 in mice treated with saline + vehicle, NTB + saline, NTB + A-68930, or NTB + SCH-23390. Values are means ± SEM (*n* = 5). **(C)** Western blot analysis of striatal levels of Cdk5-pTyr15 and total Cdk5 in mice treated with saline + vehicle, NTB + saline, NTB + quinpirole, or NTB + raclopride. Values are means ± SEM (*n* = 5). *P < 0.05 versus mice treated with NTB + saline; one-way ANOVA [*F*_(3,16)_ = 44.790] followed by Scheffe test. **(D)** Western blot analysis of striatal levels of DARPP-32-pThr75, DARPP-32-pThr34, and total DARPP-32 in mice treated with saline + vehicle, NTB + saline, NTB + quinpirole, or NTB + raclopride. ^#^*P* < 0.05 versus mice treated with NTB + saline; one-way ANOVA [*F*_(3,16)_ = 116.883] followed by Scheffe test.

To test if D1R- and D2R-mediated signals differentially affected the decrease of phosphorylation of Tyr15-Cdk5 and Thr75-DARPP-32 by nilotinib, we next performed western blotting on the striatal extracts from naïve mice injected with nilotinib (25 mg/kg) in combination with the D1-agonist A-68930 (2 mg/kg), D1-antagonist SCH-23390 (0.5 mg/kg), D2-agonist quinpirole (3 mg/kg), or D2-antagonist raclopride (1 mg/kg), 30 min before they were sacrificed. Nilotinib-induced decrease of striatal levels of Cdk5-pTyr15 (**Figure 3A**; *P* > 0.05, ANOVA) and DARPP-32-pThr75 (**Figure 3B**; *P* > 0.05, ANOVA) was unchanged by A-68930 or SCH-23390. In contrast, it was significantly enhanced by quinpirole (**Figures 3C,D**; *P* < 0.05, ANOVA), but antagonized by raclopride (**Figures 3C,D**; *P* < 0.05, ANOVA). In addition, striatal expression of total Cdk5, total DARPP-32, and DARPP-32-pThr34 was not affected by any dopaminergic compounds (**Figures 3A–D**). These findings indicate that D2R, but not D1R, activation could positively affect the nilotinib-induced decrease of striatal phosphorylation of Tyr15-Cdk5 and Thr75-DARPP-32.

### NILOTINIB SYNERGIZES WITH A D1-AGONIST FOR STRIATAL c-Fos INDUCTION IN NAÏVE MICE

**FIGURE 4:**
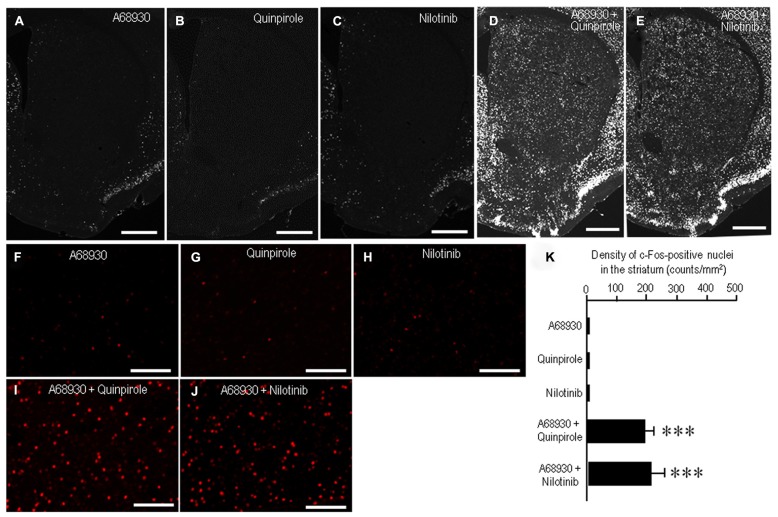
**Synergism of nilotinib and a D1-agonist for striatal c-Fos induction in naïve mice.** c-Fos immunostaining was performed on striatal sections from mice that received A-68930 (2 mg/kg) alone, quinpirole (3 mg/kg) alone, nilotinib (25 mg/kg) alone, A-68930 (2 mg/kg) + quinpirole (3 mg/kg), or A-68930 (2 mg/kg) + nilotinib (25 mg/kg) 120 min before they were sacrificed. *n* = 5 per group. **(A–J)** Photomicrographs of striatal sections stained for c-Fos from mice treated with A-68930 **(A,F)**, quinpirole **(B,G)**, nilotinib **(C,H)**, A-68930 + quinpirole **(D,I)**, and A-68930 + nilotinib **(E,J)**. Scale bar: 1 mm **(A–E)**, 100 mm **(F–J)**. **(K)** Density measurements of c-Fos-labeled nuclei in the dorsal striatum. Values are means ± SEM (*n* = 25). ***P < 0.001 versus mice treated with A-68930 alone, those with quinpirole alone, or those with nilotinib alone; unpaired two-tailed *t*-test.

A striking feature of the neuronal responses to dopamine receptor stimulation in the striatum is a rapid induction of c-Fos, one of the products of the immediate-early genes (LaHoste et al., 1993; Canales and Graybiel, 2000). Strong interactive and synergistic effects of D1Rs and D2Rs have been reported to affect these neuronal responses (LaHoste et al., 1993; Canales and Graybiel, 2000). To assess whether nilotinib could induce striatal c-Fos expression, we performed c-Fos immunostaining on striatal sections from mice that received A-68930 (2 mg/kg) alone, quinpirole (3 mg/kg) alone, nilotinib (25 mg/kg) alone, A-68930 (2 mg/kg) + quinpirole (3 mg/kg), or A-68930 (2 mg/kg) + nilotinib (25 mg/kg) at 120 min before they were sacrificed. Microscopic images showed only a few c-Fos-positive nuclei in the striatum of mice that received A-68930 alone (**Figures 4A,F**), quinpirole alone (**Figures 4B,G**), or nilotinib alone (**Figures 4C,H**). In contrast, a robust induction of c-Fos in striatal neurons was found in mice injected with the combination of A-68930 and quinpirole (**Figures 4D,I**) and in those injected with the combination of A-68930 and nilotinib (**Figures 4E,J**). These visual impressions were confirmed by densitometric analyses (**Figure 4K**). In accordance with the fact that dopamine D1/D2 synergism is required for striatal Fos induction in the intact striatum (LaHoste et al., 1993), a significant increase in the number of c-Fos-labeled nuclei was found in mice injected with the combination of A-68930 and quinpirole (**Figure 4K**; *P* < 0.001, unpaired two-tailed *t*-test), compared to mice injected with A-68930 alone. Interestingly, we also found a significant increase in the number of c-Fos-labeled nuclei in mice injected with the combination of A-68930 and nilotinib (**Figure 4K**; *P* < 0.001, unpaired two-tailed *t*-test), compared to mice injected with A-68930 alone. These findings suggest that, like a D2-agonist, nilotinib can synergize with a D1-agonist for inducing striatal c-Fos expression.

### NILOTINIB ATTENUATES MOTOR IMPAIRMENTS IN MPTP MICE

**FIGURE 5:**
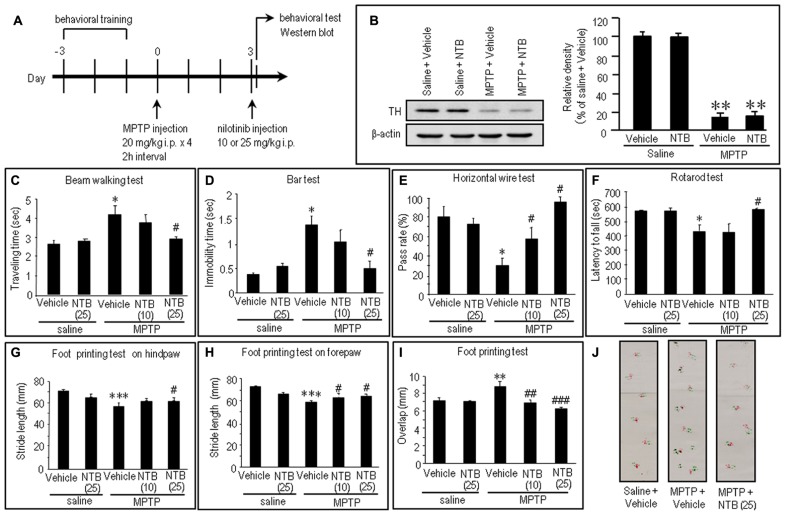
**Effects of nilotinib on MPTP-induced motor deficits in mice. (A)** Experimental design. Mice were trained for behavioral tests for three consecutive days before MPTP administration. Mice received intraperitoneal administration of MPTP (20 mg/kg × 4) or equivalent volume of saline 3 days before the behavioral study. All the behavioral tests and western blots were carried out 30 min after intraperitoneal injection of vehicle or nilotinib (NTB) at a dose of 10 or 25 mg/kg. Vehicle-treated mice received an equivalent volume of 0.5% aqueous carboxymethyl cellulose. **(B)** Western blot analysis of striatal levels of tyrosine hydroxylase (TH) in mice treated with saline + vehicle, saline + NTB, MPTP + vehicle, or MPTP + NTB. Values are means ± SEM (*n* = 5). **P < 0.01 versus mice with saline-vehicle; one-way ANOVA [*F*_(3,16)_ = 30.73] followed by Scheffe test. **(C–J)** Behavioral studies. All the values are expressed as means ± SEM. **(C)** Beam walking test (*n* = 11–14 per group). *P < 0.05 versus saline-treated mice injected with vehicle or nilotinib (25 mg/kg); ^#^*P* < 0.05 versus MPTP-treated mice injected with vehicle; one-way ANOVA [*F*_(4,61)_ = 4.31] followed by Newman–Keuls test. **(D)** Bar test (*n* = 9–10 per group). *P < 0.05 versus saline-treated mice injected with vehicle or nilotinib (25 mg/kg); ^#^*P* < 0.05 versus MPTP-treated mice injected with vehicle; one-way ANOVA [*F*_(4,43)_ = 5.97] followed by Newman–Keuls test. **(E)** Horizontal wire test (*n* = 3 per group: the number of individuals per each group was 14–19). *P < 0.05 versus saline-treated mice injected with vehicle or nilotinib (25 mg/kg); ^#^*P* < 0.05 versus MPTP-treated mice injected with vehicle; one-way ANOVA [*F*_(4,10)_ = 7.63] followed by Newman–Keuls test. **(F)** Rotarod test (*n* = 14–28 per group). *P < 0.05 versus saline-treated mice injected with vehicle or nilotinib (25 mg/kg); ^#^*P* < 0.05 versus MPTP-treated mice injected with vehicle; one-way ANOVA [*F*_(4,93)_ = 5.19] followed by Newman–Keuls test. **(G)** Foot printing test of stride length of hindpaw (*n* = 6–14 per group). ***P < 0.001 versus saline-treated mice injected with vehicle or nilotinib (25 mg/kg); ^#^*P* < 0.05 versus MPTP-treated mice injected with vehicle; one-way ANOVA [*F*_(4,43)_ = 17.24] followed by Fisher’s PLSD test. **(H)** Foot printing test of stride length of forepaw (*n* = 6–13 per group). ***P < 0.001 versus saline-treated mice injected with vehicle or nilotinib (25 mg/kg); ^#^*P* < 0.05 versus MPTP-treated mice injected with vehicle; one-way ANOVA [*F*_(4,42)_ = 17.38] followed by Fisher’s PLSD test. **(I)** Foot printing test of overlap (*n* = 6–13 per group). **P < 0.01 versus saline-treated mice injected with vehicle or nilotinib (25 mg/kg); ^##^*P* < 0.01, ^###^*P* < 0.001 versus MPTP-treated mice injected with vehicle; one-way ANOVA [*F*_(4,42)_ = 5.52] followed by Fisher’s PLSD test. **(J)** Representative images of the foot prints of saline-treated control mice, MPTP-treated mice injected with vehicle, and MPTP-treated mice injected with NTB (25 mg/kg).

To gain insight into the antiparkinsonian actions of nilotinib, we conducted an experiment in the MPTP mouse model. The experimental design is shown in **Figure 5A** (see also Materials and Methods). There was a marked (>80%) loss of TH, the rate-limiting enzyme in dopamine synthesis, in MPTP-treated mice compared to saline-treated mice (**Figure 5B**; *P* < 0.01, ANOVA). Consistent with a recent landmark report (Viaro et al., 2010), MPTP-treated mice exhibited overt behavioral abnormalities as a parkinsonian-like phenotype (**Figures 5C–J**). Compared to control mice, MPTP-treated mice showed significant motor deficits, as determined by beam walking (**Figure 5C**; *P* < 0.05, ANOVA), bar (**Figure 5D**; *P* < 0.05, ANOVA), horizontal wire (**Figure 5E**; *P* < 0.05, ANOVA), and rotarod tests (**Figure 5F**; *P* < 0.05, ANOVA). A foot printing test showed a significant decrease in stride length of both the hindpaw (**Figure 5G**; *P* < 0.001, ANOVA) and forepaw (**Figure 5H**; *P* < 0.001, ANOVA) and a significant increase in the overlap length (**Figure 5I**; *P* < 0.01, ANOVA) in MPTP-treated mice, compared to saline-treated mice.

Notably, all the behavioral tests (**Figures 5C–J**) revealed significant recovery of impaired motor performances after intraperitoneal injection of nilotinib in MPTP mice, compared to vehicle-treated MPTP mice. The beam walking (**Figure 5C**) and bar (**Figure 5D**) tests showed a significant decrease in traveling time and in catalepsy-like immobilization after administration of nilotinib at a dose of 25 mg/kg (*P* < 0.05, ANOVA), but not at a dose of 10 mg/kg (*P* > 0.05, ANOVA). The horizontal wire test (**Figure 5E**) showed a significant increase in pass rate following injection of nilotinib at doses of 10 mg/kg (*P* < 0.05, ANOVA) and 25 mg/kg (*P* < 0.05, ANOVA). The rotarod test (**Figure 5F**) showed a significant increase in latency to fall after administration of nilotinib at a dose of 25 mg/kg (*P* < 0.05, ANOVA), but not at a dose of 10 mg/kg (*P* > 0.05, ANOVA). On the foot printing test, a significant increase in stride length of the hindpaw was found after nilotinib administration at a dose of 25 mg/kg (**Figure 5G**; *P* < 0.05, ANOVA), but not at a dose of 10 mg/kg (*P* > 0.05, ANOVA), while that of the forepaw was found after administration of nilotinib at doses of 10 and 25 mg/kg (**Figure 5H**; *P* < 0.05, ANOVA). The foot printing test also showed a significant decrease in overlap length after administration of nilotinib at doses of 10 mg/kg (**Figure 5I**; *P* < 0.01, ANOVA) and 25 mg/kg (*P* < 0.001, ANOVA). These observations indicate that systemic administration of nilotinib could attenuate the motor impairments caused by striatal dopamine depletion in MPTP mice.

### NILOTINIB REVERSES ABNORMALLY INCREASED STRIATAL PHOSPHORYLATION OF Tyr15-Cdk5 and Thr75-DARPP-32 in MPTP MICE

**FIGURE 6:**
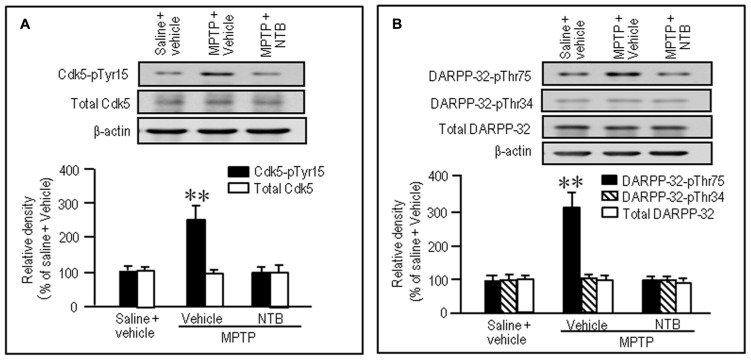
**Nilotinib affects striatal phosphorylation of Cdk5 and DARPP-32 in MPTP mice.** Saline- or MPTP-treated mice were administered vehicle or nilotinib (NTB; 25 mg/kg) 30 min before they were sacrificed. Striatal tissue extracts were prepared and subjected to western blotting. **(A)** Western blot analysis of striatal levels of Cdk5 and Cdk5-pTyr15 in mice treated with saline + vehicle (control), MPTP + vehicle, or MPTP + NTB. Values are means ± SEM (*n* = 5). **P < 0.01 versus mice with saline + vehicle or MPTP + NTB; one-way ANOVA [*F*_(2,12)_ = 11.91] followed by Scheffe test. **(B)** Western blot analysis of striatal levels of DARPP-32-pThr75, DARPP-32-pThr34, and total DARPP-32 in mice treated with saline + vehicle (control), MPTP + vehicle, or MPTP + NTB. Values are means ± SEM (*n* = 5). **P < 0.01 versus mice with saline + vehicle or MPTP + NTB; one-way ANOVA [*F*_(2,12)_ = 11.77] followed by Scheffe test.

To test if nilotinib actually inhibited striatal phosphorylation of Cdk5 and DARPP-32 in MPTP mice, we performed western blot analysis on striatal extracts from MPTP mice that received nilotinib (25 mg/kg) 30 min before they were sacrificed. A significant increase in the striatal levels of Cdk5-pTyr15 (**Figure 6A**; *P* < 0.01, ANOVA), but not of total Cdk5 (**Figure 6A**; *P* > 0.05, ANOVA), was found in MPTP mice, compared to control mice. Nilotinib treatment reversed the abnormally elevated striatal levels of Cdk5-pTyr15 (**Figure 6A**; *P* < 0.01, ANOVA) to the control baseline in MPTP mice. We also found a significant increase in striatal levels of DARPP-32-pThr75 (**Figure 6B**; *P* < 0.01, ANOVA), but not of DARPP-32-pThr34 (**Figure 6B**; *P* > 0.05, ANOVA) and total DARPP-32 (**Figure 6B**; *P* > 0.05, ANOVA), in MPTP mice compared to control mice. Nilotinib treatment reversed the abnormally elevated striatal levels of DARPP-32-pThr75 to the control baseline in MPTP mice (**Figure 6B**; *P* < 0.01, ANOVA). Thus, nilotinib could normalize the increased activity of Cdk5/DARPP-32-Thr75 signaling in the striatum of MPTP mice.

## DISCUSSION

Our results demonstrated the following four major findings: (i) nilotinib inhibited striatal phosphorylation of both Tyr15-Cdk5 and Thr75-DARPP-32 in naïve and MPTP mice, (ii) nilotinib-induced inhibition of striatal phosphorylation of Tyr15-Cdk5 and Thr75-DARPP-32 was positively affected by D2R-mediated signaling activity in naïve mice, (iii) like a D2-agonist, nilotinib synergized with a D1-agonist for inducing striatal c-Fos expression in naïve mice, and (iv) nilotinib attenuated motor impairments caused by striatal dopamine deficiency in MPTP mice. On the basis of these findings, we suggest that nilotinib might exert antiparkinsonian effects by inhibiting activity of the c-Abl/Cdk5/DARPP-32 signaling pathway in the striatum. DARPP-32 integrates the activities of dopaminergic and glutamatergic transmission in the striatum (Svenningsson et al., 2004; Fernandez et al., 2006), and is, therefore, thought to be a key regulator for the emergence of PD symptoms (Greengard, 2001). It has been suggested that a major mechanism by which dopamine and glutamate produce opposing physiological effects involves a positive feedback loop that amplifies their mutually antagonistic actions (Greengard, 2001). In such a scenario, glutamate inputs would increase phosphorylation of DARPP-32 at Thr75, the substrate site targeted by Cdk5, and thereby antagonize postsynaptic dopamine functions. Given the evidence that c-Abl can activate Cdk5 by phosphorylating its Tyr15 residue (Zukerberg et al., 2000; Dhavan and Tsai, 2001; Zhang et al., 2007), we hypothesized that nilotinib, a c-Abl inhibitor, might exert antiparkinsonian actions by inhibiting striatal phosphorylation of Tyr15-Cdk5, resulting in decreased activity of glutamate/Cdk5/DARPP-32-pThr75 signaling in the striatum. This hypothesis is supported by the fact that Cdk5-pTyr15 is a striatal-enriched phosphoprotein that is highly concentrated in the striatal matrix compartment (Morigaki et al., 2011; Yamamura et al., 2013), which has a tight link with the striatal motor functions (Crittenden and Graybiel, 2011). As in our previous study (Yamamura et al., 2013), the present study also suggests a new idea that c-Abl might be an important regulator of striatal D2R-mediated signals. However, the precise mechanism by which c-Abl activity is modulated by the interactions between dopamine and glutamate transmission in the striatum remains to be elucidated.

Current pharmacotherapy for parkinsonian motor symptoms largely depends on the activation of dopamine receptors. L-DOPA therapy still remains the gold standard for treating PD; however, after long-term exposure to L-DOPA, PD patients become refractory to treatment (Jankovic, 2005), probably a result, in part, of the down-regulation of D2Rs (Thobois et al., 2004). Moreover, patients often manifest L-DOPA-induced dyskinesias (Jankovic, 2005), a result, in part, of maladaptive synaptic plasticity at D1Rs coupled with glutamate receptors (Jenner, 2008; Murer and Moratalla, 2011). Exploration of alternative and additional therapeutic tools, particularly drugs that can exert anti-PD effects without direct activation of dopamine receptors, is therefore prudent. In this study, we provide a novel finding that in MPTP mice, striatal motor behaviors were normalized by systemic administration of nilotinib, a drug that is now clinically used to treat chronic myeloid leukemia (Weisberg et al., 2006; Blay and von Mehren, 2011). Together with the reported experimental findings that nilotinib (Hebron et al., 2013) and other c-Abl inhibitors (Ko et al., 2010; Imam et al., 2011, 2013) also play a protective role against the neurodegeneration of dopamine-producing cells in the substantia nigra of mice, our results suggest that nilotinib may serve as an alternative agent for attenuating motor symptoms and disease progression of PD.

## Conflict of Interest Statement

The authors declare that the research was conducted in the absence of any commercial or financial relationships that could be construed as a potential conflict of interest.
